# An Observational Retrospective and Prospective Study to Evaluate the Treatment Outcomes of Atlantoaxial Osteoarthritis

**DOI:** 10.7759/cureus.112340

**Published:** 2026-07-09

**Authors:** Bharatkumar R Dave, Shivanand C Mayi, Yogenkumar Adodariya, Ajay Krishnan, Ravi Ranjan Rai, Mirant B Dave, Mikeson Panthackel, Arjit Vashishtha, Saurabh S Kulkarni, Kishan Panjwani, Rushikesh B Shahade

**Affiliations:** 1 Spine Surgery, Stavya Spine Hospital and Research Institute, Ahmedabad, IND; 2 Spine, Bhavnagar Institute Of Medical Science, Bhavnagar, IND; 3 Spine, Stavya Spine Hospital, Ahmedabad, IND; 4 Orthopedics, Mahatma Gandhi Medical College and Research Institute, Aurangabad, IND

**Keywords:** atlantoaxial osteoarthritis, c1-c2 fusion, c1-c2 osteoarthritis, conservative management, occipitocervical pain

## Abstract

Background

Atlantoaxial osteoarthritis (AAOA) is a degenerative disorder of the C1-C2 articulation that causes occipitocervical pain, restricted head rotation, and functional limitation. Treatment ranges from nonoperative care to image-guided intra-articular injection and surgical stabilization in selected patients with persistent symptoms or structural compromise. This study evaluated the clinical and radiographic outcomes of patients with AAOA managed through a defined institutional treatment protocol.

Methods

This single-center observational cohort study included 232 patients with AAOA treated at a tertiary spine center. The cohort included retrospectively identified patients treated from January 2020 to March 2021 and prospectively followed patients treated from April 2021 to December 2025. All patients underwent initial nonoperative treatment according to an institutional protocol. Patients with persistent symptoms despite conservative care were offered image-guided intra-articular C1-C2 injection, and patients with refractory pain or structural compromise underwent posterior C1-C2 fusion. For analysis, patients were categorized according to definitive treatment into a nonoperative cohort and a surgical cohort. The nonoperative cohort included patients who improved with conservative treatment alone or with injection-based treatment and did not undergo surgery. Clinical outcomes were assessed using the Visual Analog Scale (VAS) and Neck Disability Index (NDI). Radiographic variables included laterality, joint type, osteoarthritis grade, periarticular ossicles, joint destruction, subluxation, instability, and fusion status in operated patients.

Results

A total of 232 patients were included, with 200 patients in the nonoperative cohort and 32 patients in the surgical cohort. All patients received initial conservative treatment, and 32 patients progressed to C1-C2 fusion. The surgical cohort was significantly younger than the nonoperative cohort, with a mean age of 50.0 ± 17.5 years compared with 65.7 ± 9.9 years, respectively. Bilateral involvement and mixed joint disease were the most common radiographic patterns in both cohorts. The surgical cohort showed greater radiographic severity, with higher rates of Grades 3 and 4 disease, joint destruction, subluxation, and instability. In the nonoperative cohort, the mean VAS score improved from 6.23 ± 1.68 at baseline to 1.61 ± 1.04 at final follow-up, and the mean NDI improved from 0.32 ± 0.11 to 0.06 ± 0.05. In the surgical cohort, the mean VAS score improved from 7.41 ± 1.19 to 1.97 ± 1.45, and the mean NDI improved from 0.60 ± 0.14 to 0.11 ± 0.10. Radiographic fusion was documented in 30 of 32 operated patients.

Conclusions

AAOA is a clinically important cause of occipitocervical pain and disability. In this single-center cohort managed through a defined institutional treatment protocol, most patients remained in the nonoperative pathway and demonstrated clinical improvement, while posterior C1-C2 fusion was associated with meaningful improvement in selected patients with refractory symptoms or structural compromise. Further prospective studies using standardized imaging, treatment protocols, and follow-up schedules are needed to refine management pathways for this condition.

## Introduction

Atlantoaxial osteoarthritis (AAOA) is a degenerative disorder involving the C1-C2 articulation and may affect the lateral atlantoaxial joints, the atlanto-odontoid articulation, or both. Because the C1-C2 complex contributes substantially to cervical rotation, degenerative involvement at this level can produce upper cervical pain, occipital or retroauricular pain, painful restriction of head rotation, and functional limitation [1]. The clinical presentation may overlap with other causes of cervicogenic headache, occipital neuralgia, and degenerative cervical pain, making careful clinical and radiographic correlation important.

Degenerative changes at the atlantoaxial joint increase with age. Halla and Hardin described atlantoaxial facet joint osteoarthritis as a distinct clinical syndrome in patients presenting with upper cervical and occipital pain [1]. Subsequent radiographic studies have shown that degenerative changes of the atlantoaxial complex become more common in older individuals, with involvement of the lateral C1-C2 joints, atlanto-dental articulation, or combined joint regions [2,3]. Although many patients may have radiographic degeneration, treatment decisions depend on the correlation between imaging findings, symptom severity, functional limitation, and structural features such as joint destruction, subluxation, or instability.

Management of AAOA is generally stepwise. Initial treatment commonly includes analgesic or anti-inflammatory medication, short-term cervical immobilization, activity modification, and physiotherapy. In patients with persistent pain despite conservative care, image-guided intra-articular injection of the C1-C2 joint may be used as an intermediate intervention for symptom control. Surgical stabilization is generally reserved for patients with refractory pain, failure of nonoperative treatment, or structural compromise such as joint destruction, subluxation, or instability [4-14].

Most available studies on AAOA are case series, surgically focused reports, injection-based studies, or narrative reviews [4-14]. As a result, there remains limited clinical literature describing outcomes across a complete institutional treatment pathway that includes conservative care, injection-based treatment, and surgical stabilization within the same cohort. In addition, clearly defining the treatment protocol is important for reproducibility and for interpreting outcomes across nonoperative and operative pathways.

The present study evaluated clinical and radiographic outcomes in patients with AAOA treated at a tertiary spine center through a defined institutional treatment protocol. The primary objective was to describe outcomes in patients managed definitively without surgery and in patients who underwent C1-C2 fusion for refractory symptoms or structural compromise. The secondary objective was to compare baseline radiographic features between the nonoperative and surgical cohorts to identify disease characteristics associated with escalation to surgical treatment.

## Materials and methods

Study design and setting

This single-center observational cohort study was conducted at Stavya Spine Hospital and Research Institute, Ahmedabad, India. The study included a retrospective component of consecutively treated patients with AAOA from January 2020 to March 2021 and a prospective observational component from April 2021 to December 2025.

The study was designed to evaluate clinical and radiographic outcomes in patients with AAOA managed through a defined institutional treatment protocol. The treatment protocol consisted of initial conservative treatment in all patients, image-guided intra-articular C1-C2 injection in selected patients with persistent symptoms, and posterior C1-C2 fusion in patients with refractory pain or structural compromise.

Ethical approval

The study protocol was reviewed and approved by the Institutional Ethics Committee of Stavya Spine Hospital and Research Institute. The protocol code was SSHRI/CS/NS/C1C2OA/SM/29/032021, and the corresponding Institutional Ethics Committee decision reference was SSHRI/CS/NS/C1C2OA/SM/33/03-21. The initial protocol planned enrollment of 200 patients. Expansion of the sample size from 200 to 232 patients was approved by the Institutional Ethics Committee through an approval/amendment dated June 6, 2025. Informed consent for treatment and use of anonymized clinical data was obtained according to institutional requirements.

Patient selection

Patients were eligible for inclusion if they were 40-90 years of age, had clinical symptoms compatible with AAOA, had radiographic evidence of C1-C2 osteoarthritis on open-mouth radiographs, were willing to participate, and were able to comply with follow-up assessment.

Patients were excluded if they had rheumatoid arthritis, a tumor, an infection, previous surgery at the craniovertebral junction, unwillingness to participate, incomplete clinical records, or absence of radiographic evidence of AAOA.

The diagnosis of symptomatic AAOA was based on correlation of clinical and radiographic findings. Clinical features included upper cervical pain, occipital or retroauricular pain, painful restriction of head rotation, localized upper cervical tenderness, and functional limitation. Radiographic diagnosis was based primarily on open-mouth radiographs, with CT and MRI obtained in patients with persistent symptoms, suspected structural compromise, or planned surgical treatment.

Institutional treatment protocol

All patients were managed according to a stepwise institutional treatment protocol. Initial treatment was nonoperative in all patients. Patients with persistent symptoms despite conservative care were considered for image-guided intra-articular C1-C2 injection. Patients with persistent disabling pain despite nonoperative treatment, recurrent symptoms after injection, joint destruction, subluxation, instability, or other structural features warranting stabilization were considered for posterior C1-C2 fusion.

For analysis, patients were categorized according to definitive treatment pathway into two groups. The nonoperative cohort included patients who improved with conservative treatment alone or with injection-based treatment and did not undergo surgery. The surgical cohort included patients who ultimately underwent posterior C1-C2 fusion after failed nonoperative treatment or because of structural compromise.

Although patients receiving conservative treatment alone and those receiving injection-based treatment were both included in the nonoperative cohort for outcome analysis, the number of patients receiving each treatment step was recorded to describe the treatment pathway.

Conservative treatment protocol

Initial conservative treatment consisted of oral nonsteroidal anti-inflammatory medication, nonopioid analgesia, short-term soft cervical collar support, activity modification, and physiotherapy.

Standard conservative treatment consisted of oral aceclofenac 100 mg twice daily for 4-8 weeks with pantoprazole 40 mg once daily and paracetamol 650 mg up to three times daily as rescue analgesia. In patients with contraindications to nonsteroidal anti-inflammatory drugs, including renal dysfunction, gastritis, anticoagulant use, cardiovascular risk, or other medical contraindications, non-NSAID analgesia was used according to comorbidity profile and treating physician judgment.

A soft cervical collar was advised to wear 12-18 hours a day for 4-8 weeks to restrict mobilization as much as possible. Physiotherapy consisted of cervical isometric exercises, gentle pain-free range-of-motion exercises, postural correction, local heat therapy, and avoidance of painful end-range rotation. High-velocity cervical manipulation was not included in the protocol. Patients were advised daily home exercises with supervised sessions two to three times weekly for 4-6 weeks.

Patients were reassessed after 4-6 weeks. Conservative treatment was considered successful when pain and functional limitation improved sufficiently for the patient to continue routine activities without escalation to injection or surgery. Failure of conservative treatment was defined as persistent disabling occipitocervical pain, inadequate improvement in Visual Analog Scale (VAS) or Neck Disability Index (NDI), recurrent symptoms interfering with activities of daily living, or patient preference for escalation after shared decision-making.

Intra-articular C1-C2 injection protocol

Patients with persistent symptoms despite conservative treatment were offered image-guided intra-articular C1-C2 injection when symptoms, examination, and radiographic findings were concordant. All injections were performed by spine surgeons under fluoroscopic as well as CT-guided navigation using O-arm guidance. Pre-procedure CT or MRI was reviewed to assess joint morphology and vertebral artery anatomy. Patients were positioned prone with the head supported in neutral or slight flexion according to imaging requirements, and strict aseptic precautions were followed.

A 22-gauge spinal needle was advanced using a posterior or posterolateral approach toward the posterior aspect of the lateral C1-C2 joint. The target was the lateral third of the joint line. After negative aspiration, iohexol 0.2-0.3 ml was injected under live imaging to confirm appropriate spread and exclude vascular or intrathecal uptake.

A 2-ml therapeutic mixture was prepared using 1 ml of lignocaine hydrochloride 2% and 1 ml of triamcinolone acetate 40 mg/ml. From this prepared mixture, only a low-volume aliquot was injected intra-articularly so that the final intra-articular volume, including contrast, did not exceed 1 ml. The remaining mixture was not injected into the joint. Injection was performed slowly and was stopped if resistance, capsular distension, or extra-articular spread was observed. No high-volume injection, hydrodilatation, or intentional capsular rupture was performed. This reflected institutional practice during the study period. Patients were monitored after the procedure for 12 hours for neurological symptoms, vascular symptoms, allergic reactions, or procedure-related pain. Repeat injection was not performed routinely.

The rationale for intra-articular injection was that the study population was defined by symptomatic radiographic AAOA, and the intended treatment target was the degenerative C1-C2 articulation itself. Dorsal ramus blockade, occipital nerve block, and radiofrequency-based procedures were not part of the institutional protocol during the study period. Therefore, this study does not compare intra-articular injection with dorsal ramus blockade or other injection targets and does not claim superiority of one injection approach over another.

Posterior C1-C2 fusion technique

Patients with persistent disabling pain despite nonoperative treatment or with structural compromise underwent posterior C1-C2 fusion under general anesthesia. Structural compromise included joint destruction, subluxation, instability, or advanced degenerative change considered likely to benefit from stabilization.

All patients were positioned prone with appropriate head fixation. Posterior exposure of C1-C2 was performed through a midline approach. The standard technique was Goel-Harms posterior C1 lateral mass-C2 pedicle/pars/laminar screw-rod fixation.

C1 fixation was performed using lateral mass screws. C2 fixation was performed using pedicle screws when anatomy permitted; pars or translaminar screws were used when required by C2 morphology or vertebral artery anatomy. Screw placement was performed under O-arm navigation guidance. Rods were contoured and secured after reduction and alignment were confirmed.

The C2 nerve root was preserved whenever possible and gently mobilized or retracted to expose the C1 lateral mass. Sacrifice of the C2 nerve root was not performed routinely and was reserved only for cases in which exposure or safe instrumentation required it. The C1-C2 joints and posterior elements were decorticated, and posterior superior iliac crest autograft was placed over the decorticated C1 posterior arch, C2 lamina, and/or C1-C2 joint region to promote arthrodesis.

Wound closure was performed in layers with a drain according to surgeon preference. Postoperatively, patients were mobilized as tolerated and were advised soft cervical collar support for four weeks according to surgeon preference, bone quality, and construct stability.

Postoperative imaging included postoperative day 1 radiographs to assess implant position, followed by radiographs at six weeks. CT was obtained between 6 and 12 months or when fusion status was uncertain or clinically indicated. Radiographic fusion was defined as bridging trabecular bone across the intended fusion area, absence of implant loosening or breakage, and absence of abnormal motion on dynamic radiographs when available.

Clinical outcome assessment

Baseline clinical evaluation included history, physical examination, neurological examination, and radiographic assessment. Clinical outcomes were assessed using the VAS for pain and the NDI for functional disability [15,16].

For the nonoperative cohort, baseline was defined as the initial clinical presentation before initiation of the institutional treatment protocol. For the surgical cohort, baseline was defined as the preoperative assessment after failure of nonoperative treatment or after the decision for surgical stabilization.

Clinical outcomes were assessed at baseline, intermediate follow-up, and final follow-up. Intermediate follow-up was defined as assessment performed between four and eight months after treatment initiation or surgery, with a target time point of six months. Final follow-up was defined as the latest available clinical assessment performed at or beyond 12 months.

The NDI was maintained and analyzed in proportional form on a 0-1 scale, with higher values indicating greater disability. When required for comparison with published literature, values were converted descriptively to a 0-100 percentage scale.

Radiographic evaluation

Radiographic evaluation for study enrollment was based on open-mouth radiographs. Computed tomography and magnetic resonance imaging were obtained in patients with persistent pain despite conservative management, suspected structural compromise, or planned surgical treatment.

Radiographic variables included laterality of involvement, joint type, osteoarthritis grade, periarticular ossicles, joint destruction, subluxation, instability, and radiographic fusion in operated cases. Laterality was classified as right, left, or bilateral. Joint involvement was classified as atlanto-odontoid, lateral C1-C2, or mixed.

Osteoarthritis severity was graded using a radiographic osteoarthritis grading framework adapted from degenerative features described by Kellgren and Lawrence [17]. This framework was applied to the C1-C2 articulation for internal severity stratification in the present cohort and was not intended to represent a separately validated C1-C2-specific grading system.

Individual radiographic findings, including periarticular ossicles, joint destruction, subluxation, and instability, were recorded separately as binary variables because these features could coexist with different overall severity grades (Table 1). Therefore, the number of patients with periarticular ossicles as an individual radiographic finding was not expected to correspond exactly to the number assigned to Grade 2 disease. Similarly, joint destruction was recorded as a separate marker of structural compromise and was not treated as equivalent to Grade 5 disease.

**Table 1:** Radiographic grading framework for C1-C2 osteoarthritis. Radiographic grading framework for C1-C2 osteoarthritis based on radiographic osteoarthritis features described by Kellgren and Lawrence [17]. C1-C2: first and second cervical vertebrae.

Grade	Radiographic feature
Grade 1	Osteophyte formation
Grade 2	Periarticular ossicles, mainly in small joints
Grade 3	Joint-space narrowing with sclerosis of subchondral bone
Grade 4	Subchondral cysts
Grade 5	Altered shape of bone ends

**Figure 1:**
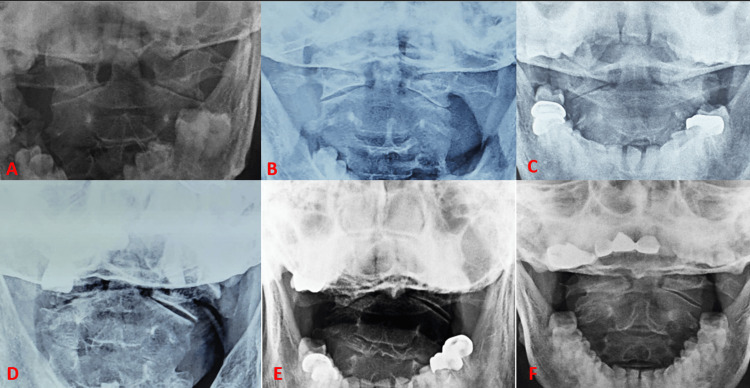
Grades of C1-C2 osteoarthritis on open-mouth radiographs. (A) Grade 1: osteophyte formation; (B) Grade 2: periarticular ossicles; (C) Grade 3: joint-space narrowing with sclerosis of subchondral bone; (D) Grade 4: subchondral cysts; (E) Grade 5: altered shape of bone ends; (F) joint destruction. C1-C2: first and second cervical vertebrae.

Representative open-mouth radiographs demonstrating radiographic findings of C1-C2 osteoarthritis are shown in Figure 1.

Radiographic grading was performed independently by two reviewers, one spine surgeon and one radiologist, using the institutional picture archiving and communication system. Disagreements were resolved by consensus, and the final decision was confirmed by the primary investigator.

Statistical analysis

Data were entered and maintained in Microsoft Excel (Redmond, Washington). Statistical analysis was performed using IBM SPSS Statistics for Windows, Version 31 (Released 2025; IBM Corp., Armonk, New York).

Continuous variables were summarized as mean ± standard deviation or median with range, as appropriate. Categorical variables were summarized as numbers and percentages.

Age was compared between the nonoperative and surgical cohorts using Welch’s t-test because of unequal group sizes and possible unequal variance. Pearson’s chi-square test was used for categorical variables only when chi-square assumptions were satisfied. These assumptions included independence of observations, mutually exclusive categories, no expected cell count less than 1, and at least 80% of expected cell counts being 5 or greater. When these assumptions were not met, Fisher’s exact test was used for 2 × 2 tables, and the Fisher-Freeman-Halton exact test or Monte Carlo exact test was used for larger contingency tables. Odds ratios were reported for 2 × 2 Fisher’s exact comparisons.

For radiographic osteoarthritis grade, Pearson’s chi-square test was performed across Grades 1-4. Grade 5 was retained in the table for descriptive completeness but excluded from inferential testing because no patient in either cohort had Grade 5 disease. The chi-square result for radiographic grade distribution was interpreted cautiously because some expected cell counts were less than 5.

Longitudinal changes in VAS and NDI scores were evaluated within each cohort across baseline, intermediate follow-up, and final follow-up using repeated-measures analysis of variance. The F-statistic was reported for the overall time effect. Statistical significance was set at p < 0.05.

Because this was a non-randomized observational study, comparisons between cohorts were interpreted as descriptive and associative rather than causal.

## Results

Study cohort and treatment pathway

A total of 232 patients with AAOA were included in the final analysis. Of these, 200 patients were included in the definitive nonoperative cohort, and 32 patients were included in the surgical cohort.

All 232 patients underwent initial conservative treatment according to the institutional treatment protocol. Among these patients, 119 underwent image-guided intra-articular C1-C2 injection for persistent symptoms after conservative treatment. Eighty-seven patients improved without further surgical intervention and remained in the nonoperative pathway, while 32 patients progressed to posterior C1-C2 fusion because of refractory symptoms or structural compromise.

For outcome analysis, patients were analyzed according to definitive treatment pathways. The nonoperative cohort included patients who improved with conservative treatment alone or with injection-based treatment and did not undergo surgery. The surgical cohort included patients who underwent posterior C1-C2 fusion.

Baseline demographic and radiographic characteristics

The surgical cohort was significantly younger than the nonoperative cohort, with a mean age of 50.0 ± 17.5 years compared with 65.7 ± 9.9 years, respectively (Welch’s t = 4.95; df = 34.24; p < 0.001).

Bilateral involvement was the most common laterality pattern in both cohorts. Laterality distribution did not differ significantly between the nonoperative and surgical cohorts (χ² = 0.34; df = 2; p = 0.846). Mixed joint involvement was also the predominant joint pattern in both groups, and joint-type distribution did not differ significantly between cohorts (Fisher-Freeman-Halton exact test, p = 0.997).

Radiographic severity differed significantly between cohorts. In the nonoperative cohort, Grade 2 disease was most frequent, followed by Grades 3, 1, and 4 disease. In the surgical cohort, Grade 3 disease was most frequent, followed by Grades 2 and 4 disease. Grade 5 disease was included in the grading framework for descriptive completeness but was not observed in either cohort. Radiographic grade distribution across Grades 1-4 differed significantly between the nonoperative and surgical cohorts (χ² = 18.98; df = 3; p < 0.001).

Markers of structural compromise were more frequent in the surgical cohort. Joint destruction, subluxation, and instability were significantly more common among surgically treated patients. Periarticular ossicles were more frequently observed in the nonoperative cohort (Table 2).

**Table 2:** Study cohort and baseline radiographic characteristics. Age was compared using Welch’s t-test. Laterality and periarticular ossicles were compared using Pearson’s chi-square test because chi-square assumptions were satisfied. Joint type was compared using the Fisher-Freeman-Halton exact test because expected cell counts were small. Joint destruction, subluxation, and instability were compared using Fisher’s exact test, and odds ratios are reported as the odds in the surgical cohort compared with the nonoperative cohort. For radiographic osteoarthritis grade, Pearson’s chi-square test was performed across Grades 1-4. Grade 5 was shown descriptively but excluded from the chi-square calculation because no Grade 5 cases were present in either cohort. The assumptions considered were independence of observations, mutually exclusive categories, and no expected cell count less than 1. The observed count for Grade 1 disease in the surgical cohort was zero; however, the expected count was approximately 4.0. As two expected cells were less than 5, the chi-square finding was interpreted cautiously as an overall comparison of grade distribution between cohorts. C1-C2: first and second cervical vertebrae; OA: osteoarthritis; OR: odds ratio; SD: standard deviation.

Variable	Nonoperative cohort (n = 200)	Surgical cohort (n = 32)	Test statistic	p-value
Age (years), mean ± SD	65.7 ± 9.9	50.0 ± 17.5	Welch’s t = 4.95; df = 34.24	<0.001
Laterality, n (%)			χ² = 0.34; df = 2	0.846
Right	40 (20.0)	6 (18.8)		
Left	41 (20.5)	8 (25.0)		
Bilateral	119 (59.5)	18 (56.2)		
Joint type, n (%)			Fisher-Freeman-Halton exact test	0.997
Atlanto-odontoid	26 (13.0)	4 (12.5)		
Lateral C1-C2	25 (12.5)	4 (12.5)		
Mixed	149 (74.5)	24 (75.0)		
Radiographic OA grade, n (%)			χ² = 18.98; df = 3	<0.001
Grade 1	29 (14.5)	0 (0.0)		
Grade 2	98 (49.0)	8 (25.0)		
Grade 3	55 (27.5)	16 (50.0)		
Grade 4	18 (9.0)	8 (25.0)		
Grade 5	0 (0.0)	0 (0.0)	Not included in test because of zero observations	Not applicable
Periarticular ossicles, n (%)	136 (68.0)	13 (40.6)	χ² = 9.00; df = 1	0.003
Joint destruction, n (%)	18 (9.0)	12 (37.5)	Fisher’s exact test; OR = 6.07	<0.001
Subluxation, n (%)	5 (2.5)	7 (21.9)	Fisher’s exact test; OR = 10.92	<0.001
Instability, n (%)	2 (1.0)	14 (43.8)	Fisher’s exact test; OR = 77.00	<0.001

Longitudinal clinical outcomes

Serial clinical assessment demonstrated marked improvement in both pain and disability in the nonoperative and surgical cohorts (Table 3).

**Table 3:** Clinical outcomes by treatment cohort. Intermediate follow-up was defined as the clinical assessment performed between four and eight months after treatment initiation or surgery, with a target time point of six months. Final follow-up was defined as the latest available clinical assessment performed at or beyond 12 months. Longitudinal comparisons were performed using repeated-measures analysis of variance within each cohort. The F-statistic represents the overall time effect. NDI: Neck Disability Index; SD: standard deviation; VAS: Visual Analog Scale.

Outcome measure	Baseline, mean ± SD	Intermediate follow-up, mean ± SD	Final follow-up, mean ± SD	Test statistic	Overall p-value
Nonoperative cohort (n = 200)					
VAS	6.23 ± 1.68	3.38 ± 1.41	1.61 ± 1.04	F = 553.28	<0.001
NDI	0.32 ± 0.11	0.17 ± 0.08	0.06 ± 0.05	F = 486.67	<0.001
Surgical cohort (n = 32)					
VAS	7.41 ± 1.19	4.31 ± 1.28	1.97 ± 1.45	F = 138.62	<0.001
NDI	0.60 ± 0.14	0.28 ± 0.11	0.11 ± 0.10	F = 142.50	<0.001

In the nonoperative cohort, mean VAS improved from 6.23 ± 1.68 at baseline to 3.38 ± 1.41 at intermediate follow-up and 1.61 ± 1.04 at final follow-up. Mean NDI improved from 0.32 ± 0.11 at baseline to 0.17 ± 0.08 at intermediate follow-up and 0.06 ± 0.05 at final follow-up. The overall reduction across time was significant for both VAS and NDI.

In the surgical cohort, baseline pain and disability were higher. Mean VAS improved from 7.41 ± 1.19 at baseline to 4.31 ± 1.28 at intermediate follow-up and 1.97 ± 1.45 at final follow-up. Mean NDI improved from 0.60 ± 0.14 at baseline to 0.28 ± 0.11 at intermediate follow-up and 0.11 ± 0.10 at final follow-up. These longitudinal improvements were also significant for both VAS and NDI.

Although the nonoperative cohort reached slightly lower final absolute VAS and NDI values, the surgical cohort started with substantially worse baseline pain and disability, indicating a higher initial disease burden.

Operative and radiographic outcomes in the surgical cohort

All 32 patients in the surgical cohort underwent C1-C2 fusion. All procedures were performed in the prone position. The mean operative time was 151.3 ± 21.6 minutes, with a range of 115-185 minutes. Radiographic fusion was documented in 30 of 32 patients (93.75%) at final follow-up (Table 4).

**Table 4:** Operative and radiographic outcomes in the surgical cohort. Operative and radiographic outcomes are shown for patients who underwent C1-C2 fusion. C1-C2: first and second cervical vertebrae; SD: standard deviation.

Variable	Surgical cohort (n = 32)
Patients undergoing C1-C2 fusion, n (%)	32 (100.0)
Prone position, n (%)	32 (100.0)
Operative time (minutes), mean ± SD	151.3 ± 21.6
Operative time range (minutes)	115-185
Radiographic fusion documented, n (%)	30 (93.75)

## Discussion

This single-center observational cohort describes the clinical and radiographic outcomes of patients with AAOA managed through a defined institutional treatment protocol. The principal findings were that most patients remained in the nonoperative pathway and demonstrated substantial improvement in pain and disability, while patients selected for posterior C1-C2 fusion had greater baseline pain, higher disability, and more frequent radiographic markers of structural compromise. Surgical treatment was associated with meaningful improvement in VAS and NDI scores, with radiographic fusion documented in 30 of 32 operated patients.

AAOA is a distinct degenerative disorder of the C1-C2 articulation that can produce upper cervical, occipital, and retroauricular pain with painful limitation of head rotation. Halla and Hardin described atlantoaxial facet joint osteoarthritis as a recognizable clinical syndrome in patients presenting with upper cervical and occipital pain [1]. Subsequent radiographic studies have shown that degenerative changes of the atlantoaxial complex become more common with advancing age and may involve the lateral C1-C2 joints, the atlanto-dental articulation, or both [2,3]. In the present cohort, mixed joint involvement and bilateral disease were the most common radiographic patterns in both treatment groups, supporting the concept that AAOA often affects more than one component of the atlantoaxial articulation.

The present study adds to existing literature by describing outcomes across a complete institutional treatment pathway rather than focusing exclusively on surgical cases. Published literature includes narrative reviews, nonoperative treatment descriptions, injection-based studies, and surgically focused series [4-14]. In contrast, the present cohort included patients managed definitively without surgery as well as those who progressed to fusion. This distinction is clinically important because AAOA exists across a spectrum of severity. Patients with less destructive disease may improve with nonoperative care, whereas patients with persistent disabling pain or structural compromise may require stabilization.

In the nonoperative cohort, mean VAS score improved from 6.23 ± 1.68 at baseline to 1.61 ± 1.04 at final follow-up, and mean NDI improved from 0.32 ± 0.11 to 0.06 ± 0.05. These findings suggest that a substantial proportion of symptomatic patients can improve without surgery when managed through a structured pathway that includes medication, cervical support, physiotherapy, and selective image-guided injection. However, because conservative-only and injection-treated patients were not analyzed as separate outcome groups, the present study cannot determine the independent effect of each nonoperative treatment component.

Image-guided intra-articular C1-C2 injection was used as an intermediate treatment step for patients with persistent symptoms despite initial conservative care. The rationale for intra-articular injection in this cohort was that the pain generator was considered to be the degenerative C1-C2 articulation itself, based on clinical and radiographic correlation. Previous studies have reported the use of lateral atlantoaxial intra-articular steroid injection in selected patients with mechanical, inflammatory, or cervicogenic pain patterns [5-7]. In the present study, intra-articular injection was part of the institutional protocol and was not compared with dorsal ramus blockade, occipital nerve block, or radiofrequency-based procedures. Therefore, no conclusion can be drawn regarding the superiority of intra-articular injection over dorsal ramus or other extra-articular injection targets.

The surgical cohort differed from the nonoperative cohort in several important ways. Surgically treated patients were younger, had higher baseline pain and disability scores, and had more frequent radiographic markers of advanced disease, including Grade 3 and Grade 4 osteoarthritis, joint destruction, subluxation, and instability. These findings indicate that the surgical cohort represented a severity-selected subgroup rather than a comparable parallel treatment arm. Therefore, the better final absolute scores in the nonoperative cohort should not be interpreted as evidence that nonoperative treatment was superior to surgery. Instead, the data suggest that treatment escalation was associated with baseline disease burden, refractory symptoms, and structural compromise.

The operative outcomes in the present study are consistent with previously published surgical series. Published reports and pooled analyses have shown that posterior C1-C2 fusion may provide substantial pain relief, functional improvement, and high fusion rates in selected patients with refractory AAOA [8-14]. In the present surgical cohort, mean VAS score improved from 7.41 ± 1.19 to 1.97 ± 1.45, and mean NDI improved from 0.60 ± 0.14 to 0.11 ± 0.10, which is directionally consistent with these prior reports.

Radiographic fusion was documented in 30 of 32 operated patients, corresponding to a fusion rate of 93.75%. This is comparable to fusion rates reported in modern surgical literature on C1-C2 arthrodesis for degenerative atlantoaxial disease [9-12]. However, the surgical cohort in the present study was relatively small, and fusion assessment was based on institutional follow-up imaging rather than a uniform prospective imaging schedule for all patients. Therefore, the fusion result should be interpreted as a descriptive surgical outcome rather than as a definitive estimate of fusion probability.

The radiographic grading framework used in this study was intended for internal severity stratification. It was adapted from established degenerative osteoarthritis radiographic features described by Kellgren and Lawrence [17]. However, it should not be interpreted as a separately validated C1-C2-specific grading system. Grade 5 disease was included for descriptive completeness but was not observed in either cohort. The absence of Grade 5 disease did not alter the study interpretation, because the main radiographic distinction between cohorts was the higher frequency of Grades 3 and 4 disease and the greater prevalence of structural compromise in the surgical group. Recording periarticular ossicles, joint destruction, subluxation, and instability as separate binary variables helped distinguish overall radiographic severity from specific structural features that may influence treatment selection.

Taken together, the findings support a pragmatic severity-based approach to AAOA. Patients without major structural compromise may improve through nonoperative care, with image-guided intra-articular injection used selectively for persistent symptoms. Patients with refractory pain, joint destruction, subluxation, or instability may achieve meaningful improvement after posterior C1-C2 fusion. Because treatment allocation was not randomized, these findings should be interpreted as descriptive outcomes within an institutional treatment pathway rather than evidence of comparative treatment superiority.

Limitations

This study has several limitations. It was an observational, non-randomized study, and treatment selection was based on symptom severity, radiographic findings, response to previous treatment, and clinical judgment. Therefore, comparisons between the nonoperative and surgical cohorts should be interpreted as descriptive and not as proof that one treatment was superior to the other. The nonoperative cohort included both patients who improved with conservative treatment alone and those who improved after intra-articular injection; because these subgroups were not analyzed separately, the individual effect of each nonoperative treatment cannot be determined. Intra-articular C1-C2 injection was part of the institutional protocol, but it was not compared with dorsal ramus block, occipital nerve block, radiofrequency ablation, or other interventional pain procedures. Therefore, this study cannot establish the superiority of one injection target over another. Advanced imaging was performed more often in patients with persistent symptoms and in those considered for surgery, which may have increased the detection of joint destruction, subluxation, and instability in the surgical cohort. The radiographic grading system was adapted from general osteoarthritis grading principles and has not been externally validated specifically for C1-C2 osteoarthritis. The surgical cohort was relatively small and represented a severity-selected group, which limits the generalizability of operative outcomes. Follow-up was performed within predefined clinical windows rather than at identical time points for every patient. Finally, this was a single-center study based on one institutional treatment protocol, and the findings may not be directly applicable to centers using different conservative regimens, injection techniques, surgical methods, or follow-up protocols.

## Conclusions

AAOA is a clinically important cause of occipitocervical pain and functional disability. In this single-center observational cohort, most patients remained in the nonoperative pathway and showed significant improvement in pain and disability after treatment through a defined institutional protocol. Patients who required posterior C1-C2 fusion had greater baseline disease severity and structural compromise, and fusion was associated with meaningful clinical improvement in this selected subgroup. These findings support a stepwise, severity-based approach to management, while further prospective studies with standardized imaging, intervention protocols, and follow-up schedules are needed to refine treatment selection.
